# 3-O-acetyl-11-keto-β-boswellic acid exerts anti-tumor effects in glioblastoma by arresting cell cycle at G2/M phase

**DOI:** 10.1186/s13046-018-0805-4

**Published:** 2018-07-03

**Authors:** Wan Li, Jinyi Liu, Weiqi Fu, Xiangjin Zheng, Liwen Ren, Shiwei Liu, Jinhua Wang, Tengfei Ji, Guanhua Du

**Affiliations:** 1 The State Key Laboratory of Bioactive Substance and Function of Natural Medicines, Beijing, 100050 China; 2 Key Laboratory of Drug Target Research and Drug Screen, Institute of Materia Medica, Chinese Academy of Medical Science and Peking Union Medical College, Beijing, 100050 China; 3 Ethnic Drug Screening & Pharmacology Center, Key Laboratory of Chemistry in Ethnic Medicinal Resources, State Ethnic Affairs Commission & Ministry of Education, Yunnan Minzu University, Kunming, 650500 China; 4 Department of Endocrinology, Shanxi DAYI Hospital, Shanxi Medical University, Taiyuan, 030002 Shanxi China

**Keywords:** Glioblastoma, AKBA, Apoptosis, Cell cycle, p21/FOXM1/cyclin B1

## Abstract

**Background:**

Glioblastoma (GBM) is the most common, malignant, and lethal primary brain tumor in adults accounting for about 50% of all gliomas. Up to now, the chemotherapy approaches for GBM were limited. 3-O-acetyl-11-keto-β-boswellic acid (AKBA), the major active ingredient of the gum resin from *Boswellia serrata* and *Boswellia carteri* Birdw., was reported to inhibit the growth of many types of cancer cells; however, the underlying mechanism of its anticancer effects are still unclear.

**Methods:**

The effects of AKBA on cell viability and its cytotoxicity were determined using CCK8 and LDH kits respectively. The EdU-DNA synthesis assay was used to evaluate inhibition of cell proliferation by AKBA. The role of AKBA in glioblastoma cell functions such as migration/invasion, and colony formation was evaluated using transwell chambers and soft agar, respectively. Flow cytometry and western blotting were used to detect AKBA-induced apoptosis. Potential mechanisms of AKBA action were explored by RNA sequencing and the identified hub genes were validated by real-time quantitative PCR and western blotting. Finally, the in vivo anti-tumor activity of AKBA was evaluated against a human glioblastoma cell line, U87-MG, in a xenograft mouse model.

**Results:**

AKBA inhibited cell proliferation, caused the release of LDH, decreased DNA synthesis, and inhibited the migration, invasion, and colony formation of U251 and U87-MG human glioblastoma cell lines. AKBA increased apoptosis as well as the activity of caspase 3/7 and the protein expression of cleaved-caspase 3 and cleaved PARP, while decreasing mitochondrial membrane potential. RNA-sequencing analyses showed that AKBA suppressed the expression of pRB, FOXM1, Aurora A, PLK1, CDC25C, p-CDK1, cyclinB1, Aurora B, and TOP2A while increasing the expression of p21 and GADD45A. These findings were validated by qRT-PCR and western blotting. The data are consistent with a mechanism in which AKBA arrested the cell cycle in glioblastoma cells at the G2/M phase by regulating the p21/FOXM1/cyclin B1 pathway, inhibited mitosis by downregulating the Aurora B/TOP2A pathway, and induced mitochondrial-dependent apoptosis. Oral administration of AKBA (100 mg/kg) significantly suppressed the tumorigenicity of U87-MG cells in a xenograft mouse model.

**Conclusions:**

Taken together, these results suggest that AKBA (molecular weight, 512.7 Da) might be a promising chemotherapy drug in the treatment of GBM.

**Electronic supplementary material:**

The online version of this article (10.1186/s13046-018-0805-4) contains supplementary material, which is available to authorized users.

## Background

Glioblastoma, accounting for about 50% of all gliomas, is the most common, malignant, and lethal primary brain tumor in adults. Despite optimized treatment strategies, the median survival for patients with GBM is 15 to 20 months from the time of diagnosis, and only 3 to 5% of patients survive longer than 5 years [1, 2]. The standard treatment includes maximal surgical resection, radiation, and chemotherapy with temozolomide (TMZ) [3, 4]. TMZ combined with radiation increases the survival time by about 3 months compared to radiotherapy alone [5, 6] and increases the 2-year survival rate from 10 to 26% [5]. However, acquired resistance, low response rate, and side effects limit TMZ’s effectiveness; therefore, mores studies on developing new chemotherapy drugs are essential.

Natural products are an important source of drugs against various human diseases including cancers [7–9] with approximately half of the pharmaceuticals being developed from plants. More novel drugs with high efficiency and low toxicity still need to be discovered [10, 11]. 3-O-acetyl-11-keto-β-boswellic acid (AKBA), isolated from the gum resin of *Boswellia serrata* and *Boswellia carteri* Birdw., is widely used in Africa, India, and China [12] to treat inflammatory diseases including arthritis [13], colitis [14], Crohn’s disease [15] and asthma [16, 17], as well as some other illnesses [18, 19]. Boswellic acid exerts its anti-inflammatory therapeutic effects by directly interacting with IκB kinases [20] and inhibiting nuclear factor-κB-regulated gene expression [21]. In addition, boswellic acid has been reported to noncompetitively inhibit 5-lipoxygenase [22, 23], topoisomerase [24], and leukocyte elastase [25]. Recent studies have shown that AKBA can induce apoptosis in several types of cancer cells including prostate [26], colon [27] and glioblastoma [28] by activating caspase-8 [29] and regulating the death receptor 5-mediated signal pathway [30]. However, whether AKBA can inhibit the growth of glioblastoma cells and what its mechanism might be are still not clear.

Here, we investigated the anti-glioblastoma effects of AKBA and found that it inhibited the viability and proliferation of the human glioblastoma cell lines, U251 and U87-MG. In addition, AKBA inhibited the migration, invasion, and colony formation of the glioblastoma cells as well as inducing them to undergo mitochondrial-dependent apoptosis. Using RNA-sequencing and western blotting analyses, we also found that AKBA arrested the cell cycle at the G2/M phase by regulating the p21/FOXM1/cyclin B1 pathway and inhibited mitosis of glioblastoma cells by downregulating the Aurora B/TOP2A pathway. Our results suggest that AKBA might be a promising chemotherapeutic drug in the treatment of GBM.

## Methods

### Cell culture

The human glioblastoma cells, U251 and U87-MG, were obtained from the Cell Bank of the Chinese Academy of Sciences (Beijing, China). Cells were maintained in Dulbecco’s Modified Eagle’s Medium (DMEM) with 10% FBS (5% CO_2_, 37 °C) and cultured according to the protocol.

### Chemotherapeutic drug

AKBA was kindly provided by Prof. TengfeiJi (Institute of Materia Medica, CAMS & PUMC) as a pure, colorless, crystalline compound that was dissolved in dimethylsulfoxide (DMSO, Sigma-Aldrich) as a stock solution of 30 mM.

### Lactate dehydrogenase (LDH) detection

LDH released from apoptotic cells or dead cells was measured using a Cytotoxicity LDH Assay Kit (Dojindo, Japan) according to manufacturer’s instructions. Cells were seeded in 96-well plates at 1 × 10^5^ cells per well and cultured for 24 h. Cells were then treated with AKBA for 24 and 48 h at a final concentration of 10, 20, and 30 μM in DMEM supplemented with 5% FBS. After AKBA treatment, 100 mL of working solution containing water-soluble tetrazolium salt was added to each well and the plate was incubated at room temperature for 30 min. Stop solution (50 μL) was added to each well and absorbance at 490 nm was measured using a SpectraMax M5 spectrophotometer (Molecular Devices, Sunnyvale, CA).

### Cell proliferation assay

The effect of AKBA on cell proliferation was assessed by counting viable cells using a colorimetric assay in the Cell Counting Kit-8 (CCK-8, Beyotime, China). Cells were seeded in 96-well plates at 3 × 10^3^ per well for 24 h before AKBA was added at concentrations of 0 to 100 μM for 24, 48, and 72 h. The absorbance at 450 nm was measured by SpectraMax M5 spectrophotometer (Molecular Devices, Sunnyvale, CA) and IC_50_ values were calculated using GraphPad Prism 7 software (GraphPad Software Inc., San Diego, California, USA).

### EdU-DNA synthesis assay

DNA synthesis activity in AKAB-treated cells was studied using the EdU (5-ethynyl-2′-deoxyuridine, EdU)-DNA synthesis assay, Cell-Light™EdU Apollo®567 In Vitro Imaging Kit (RiboBio, Guangzhou, China) according to the supplier’s protocol. Cells were seeded in 96-well plates at 1 × 10^4^ per well. After 24 h incubation, 0, 10, 20, and 30 μM AKBA were added to the cells and incubation continued for 24 and 48 h. EdU was then added to the culture media at 50 μM and after 2 h, the cells were fixed in 4% paraformaldehyde for 30 min, permeated with 0.5% Trixon-X 100 for 10 min, and stained with 10 μM Apollo 567 for 30 min. Cells were then counterstained with Hoechst 33,342 for 30 min and imaged by high content imaging system (Cellomics ArrayScan VTI, Thermo Fisher Scientific, Carlsbad, CA).

### Migration and invasion assays

Cell migration and invasion ability were measured using 24-well plates with transwell inserts with 8 μm pores (Corning Costar, USA). For the invasion assay, the well bottom was coated with 12.5% Matrigel (Corning Biocoat, USA). Cells were harvested and suspended in FBS-free culture medium. AKBA was added to culture medium at a final concentration of 10, 20 or 30 μM, and 300 μL of cell suspension (5 × 10^4^ cells/mL) was added to the upper chamber. At the same time, 1 mL of DMEM supplemented with 10% FBS was added to the lower chamber. After incubation for 24 h, cells that had passed through the membrane were fixed in 4% paraformaldehyde for 20 min, stained with 1% crystal violet for 15 min, and counted using a light microscope (Nikon, Japan).

### Soft agar colony formation assay

The soft agar colony formation assay was carried out in 6-well plates containing a bottom layer of 2 mL of 0.7% agar in DMEM supplemented with 10% FBS with a top layer of 1 mL of 0.35% agar in DMEM, 10% FBS. The plates containing 3000 cells were then incubated at 37 °C for 2–3 weeks, after which the colonies were stained with MTT (3-(4,5-dimethylthiazol-2-yl)-2,5-diphenyltetrazolium bromide, 200 μL/well) and numbers of colonies were counted.

### Apoptosis assay by Annexin V-FITC staining and flow cytometry

Viable, early and late apoptotic and necrotic cells were classified by flow cytometry (BD FACSVerse, San Diego, CA, USA) using an Annexin V-FITC apoptosis detection kit (KeyGenBioTECH, Nanjing, China). Cells were treated with 0, 10, 20, and 30 μM AKBA for 24 and 48 h, harvested, washed with PBS, and stained with Annexin V-FITC and propidium iodide for 20 min followed by flow cytometry [31].

### Measurement of mitochondrial membrane potential

5,5′,6,6′-tetrachloro-1,1′,3,3′-tetraethylbenzimidazolocarbocyanine iodide (JC-1, Beyotime, Guangzhou, China) a selectively membrane-permeant dye, was used to measure the mitochondrial membrane potential and differentiate apoptotic cells from control cells [32]. Cells were seeded in 6-well plates and treated with 0, 10, 20 and 30 μM AKBA for 24 and 48 h. After treatment, cells were harvested, washed with PBS, and incubated with JC-1 at 37 °C for 30 min. Flow cytometry analysis was then performed to determine the number of cells with altered mitochondrial membrane potential.

### Caspase 3/7 activity detection

To assess caspase activity, U251 and U87-MG cells were seeded in 6-well plates and incubated with 0, 10, 20, and 30 μM AKBA for 24 and 48 h. After treatment, cells were harvested, washed with PBS, and incubated for 1 h with reagent for Caspase 3/7 from the Live-cell Fluorescence Real-time Detection Kit (KeyGenBioTECH, Nanjing, China). Caspase 3/7 activity was analyzed by flow cytometry (BD Accuri C6, San Diego, CA, USA).

### Cell cycle assay

Cells were seeded in 6 cm dishes at 2 × 10^5^ cells, and serum-starved for 24 h before treatment with 0, 10, 20 and 30 μM AKBA for 24 and 48 h. Cells were harvested, fixed in 70% ethanol at 4 °C overnight, and then incubated with RNase and the DNA-interacting dye propidium iodide (PI) for 30 min at 37 °C. Cell cycle analysis was performed using a flow cytometer (BD Accuri C6, San Diego, CA, USA).

### Quantitative real-time PCR

Total RNA was extracted from cells with TRIzol Reagent (Life Technologies, USA) and cDNA was synthesized using the PrimeScript RT Reagent Kit (TaKaRa Clontech, Dalian, China). Real-time RT-PCR (CFX 96, thermocycler, Bio-Rad, Hercules, CA) was performed to detect the expression of differentially expressed genes using the SYBR Premix Ex TaqIIKit (TaKaRa Clontech, Dalian, China). GAPDH was used as a loading control. The primer sequences are listed in Additional file 1: Table S1.

### RNA preparation and next-generation sequencing

Cells seeded in 6-well plates at 2 × 10^5^ cells/well were treated with 20 μM AKBA for 48 h, then disrupted with TRIzol Reagent (Life Technologies, USA) and sent to CapitalBio Corporation (Beijing, China) for next-generation sequencing. RNA was qualified using the Bioanalyzer 2100 and libraries were established with NEBNext Multiplex Oligos. The libraries were normalized, pooled and sequenced using the IlluminaHiSeq™ 2500 sequencing platform (Illumina Inc., San Diego, CA, USA).

### Identification of differentially expressed genes

FASTQ data were aligned to the genome (GRh38) using HISAT2. After removing duplicate reads, the remaining SAM files were converted and sorted into BAM files using the SAMtools software [33]. Cufflinks software [34] was used to analyze differential gene expression with the following screening criteria: fold change (FC) ≥ 3 and false discovery rate (FDR) < 0.01.

### Gene ontology and pathway enrichment analyses

Gene ontology and pathway enrichment analyses were performed on the DAVID (Database for Annotation, Visualization and Integrated Discovery) bioinformatics website, v. 6.8 (https://david.ncifcrf.gov/home.jsp). The gene ontology terms were recruited from the GO dataset and the biological pathways from the KEGG datasets. The cutoff values of the enrichment results were set as: count ≥3, fold change ≥3, and EASE score < 0.05. Co-expression modules were visualized with Cytoscape 3.6.0.

### Western blotting

Cells were lysed and total protein extracted in RIPA lysis buffer (Applygen, Beijing, China) supplemented with protease inhibitors (Roche, Indianapolis, IN) and phosphatase inhibitors (Applygen, Beijing, China) at 4 °C for 30 min. The cell lysate was centrifuged at 12,500 rpm at 129,50 g-force for 15 min at 4 °C and protein concentrations were measured by BCA method (Beyotime, Guangzhou, China). Proteins were separated by 10% SDS-PAGE and transferred to a PVDF membrane (Millipore, Billerica, MA). After blocking with 5% fat-free milk in TBST for 1 h, membranes were immunoblotted with primary antibodies to caspase3, cleaved-caspase3, PARP, cleaved-PARP, p21, p-RB, PLK1, p-CDK1, and Aurora B (Cell Signaling Technology); p53, FOXM1, Aurora A, CDC-25C, CDK-1, cyclin B1, TOP2A, and GAPDH (Proteintech) at the appropriate dilutions with gentle shaking overnight at 4 °C. Blots were incubated with goat anti-mouse or anti-rabbit HRP-conjugated secondary antibody (1:3000; CWBIO, Beijing, China) and bands were visualized using an enhanced chemiluminescence, eECL Western Blot Kit (CWBIO, Beijing, China).

### Mouse xenograft tumor model and antitumor assay

All animal experiments were conducted according to the principles of the NIH Guide for the Care and Use of Laboratory Animals and were approved by the ethics committee for laboratory animal care and use of the Institute of Materia Medica, CAMS & PUMC. Six week-old female BALB/c-nu nude mice (Beijing Vital River Laboratory Animal Technology Co., Ltd., Beijing, China) weighing 15–18 g were used in this study and all mice were housed in barrier facilities with a 12 h light/dark cycle. Human U87-MG glioblastoma cells (1 × 10^7^ cells) were subcutaneously injected into the right flanks of the mice. After 20 days, when the tumor volume reached about 100 mm^3^, the mice were randomly divided into two groups (*n* = 8) and treated once daily for 14 days with AKBA (100 mg/kg body weight, p.o.) prepared in 0.5% carboxymethylcellulose sodium or vehicle control. The mice were weighed and tumor size was measured every other day. The tumor volume (V) in mm^3^ was calculated using the following formula: V = 0.5 × a × b^2^, where *a* was the length (mm), *b* was the width (mm), and V was the volume of the tumor. At the end of the experiment, all the mice were euthanized and the tumor weights and organ weights were recorded.

### Statistical analysis

The results are given as mean ± SD. One-way ANOVA was used to calculate differences between the various study groups. *P* < 0.05 was considered statistically significant.

## Results

### AKBA reduced viability and inhibited proliferation of human glioblastoma cells

**Fig. 1:**
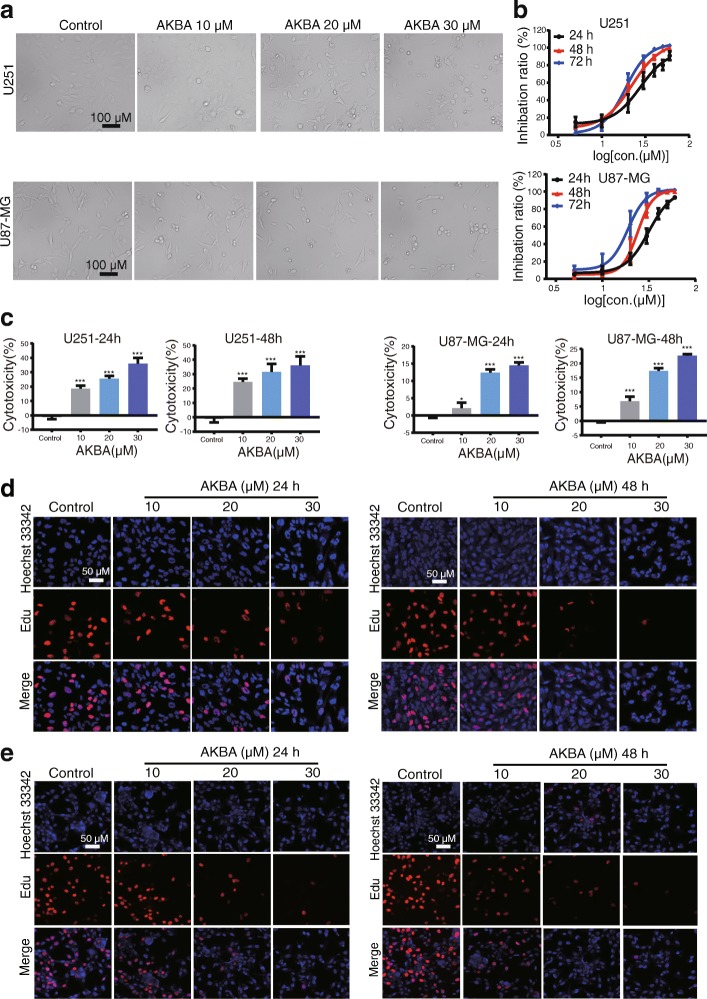
AKBA inhibits cell proliferation and increases cytotoxicity in a dose- and time-dependent manner in U251 and U87-MG cells. **a** Morphology of cells treated with AKBA at 10, 20, and 30 μM. Scale bar = 100 μm. **b** CCK8 assay shows that AKBA inhibits proliferation of U251 and U87-MG cells. **c** LDH assay shows that AKBA is cytotoxic to U251 and U87-MG cells. **d** EdU-DNA synthesis assay sho ws that AKBA inhibits DNA synthesis in U251 cells. Scale bar = 50 μm. **e** EdU-DNA synthesis assay shows that AKBA inhibits DNA synthesis in U87-MG cells. Scale bar = 50 μm. The experiments were performed in triplicate, and the data are presented as mean ± SD, *P < 0.05 vs. control group

AKBA inhibited the growth of U251 and U87-MG cells in a time- and dose-dependent manner (Fig. 1a). The IC_50_ values for AKBA on U251 cells at 24, 48, and 72 h were 27.62 μM, 22.11 μM, and 18.69 μM; and for U87-MG cells, 31.61 μM, 23.96 μM, and 18.79 μM at 24, 48, and 72 h (Fig. 1b) respectively. Therefore, we selected concentrations of 10, 20, and 30 μM AKBA to further investigate its anticancer effects and mechanism. When cells undergo apoptosis and death, their membranes are disrupted and the enzyme lactate dehydrogenase (LDH) is released into the culture medium. Results of LDH assay showed that AKBA significantly increased the LDH level in U251 and U87-MG cell culture medium in a dose- and time-dependent manner (Fig. 1c). To confirm the inhibitory effects of AKBA on cell proliferation, an EdU-DNA synthesis assay was performed. Treatment with 10, 20, and 30 μM AKBA for 24 and 48 h resulted in a dose- and time-dependent inhibition of proliferation of U251(Fig. 1d and Additional file 1: Figure. S1A) and U87-MG cells (Fig. 1e and Additional file 1: Figure S1B). Thus AKBA suppressed viability and proliferation of human glioblastoma cells.

### AKBA inhibited migration, invasion, and colony formation of glioblastoma cells

**Fig. 2:**
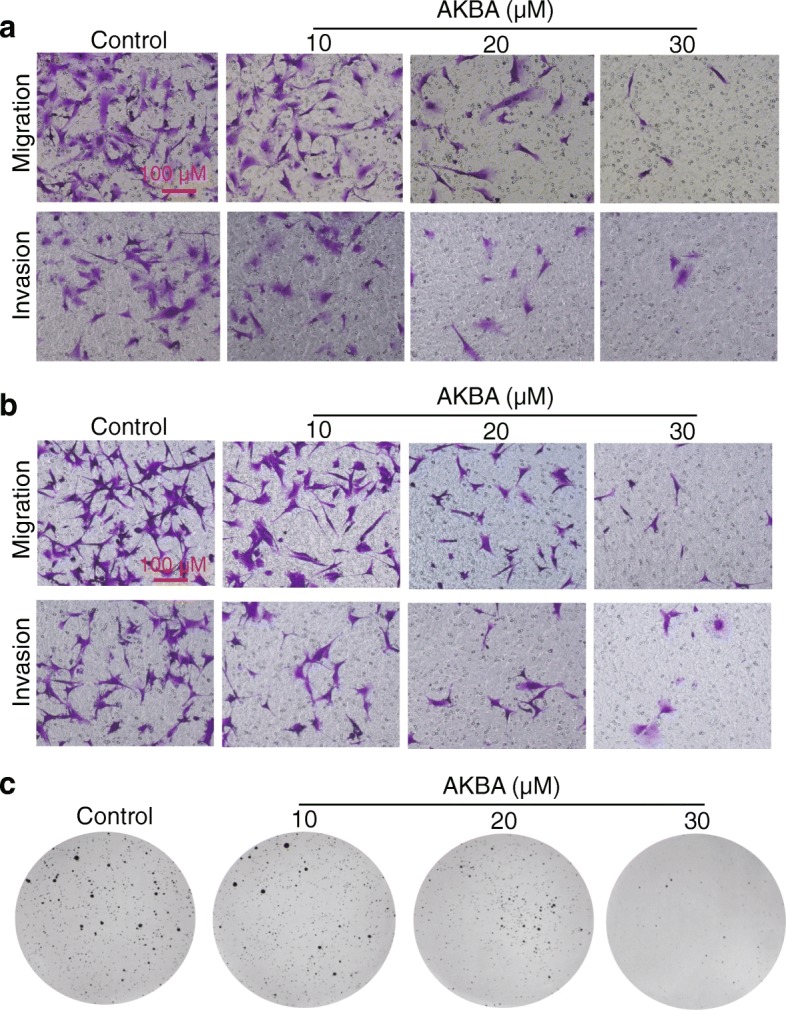
AKBA inhibits migration, invasion and colony formation in U251 and U87-MG cells in a dose-dependent manner. **a** Transwell assays show that AKBA inhibits the migration and invasion of U251 cells. **b** Transwell assays show that AKBA inhibits the migration and invasion of U87-MG cells. **c** Soft agar assays show that AKBA reduces the colony formation of U87-MG cells. The experiments were performed in triplicate, and scale bar = 100 μm

Transwell migration and invasion assays revealed that AKBA significantly inhibited the migration and invasion of U251 and U87-MG cells in a dose-dependent manner (Fig. 2a and b; Additional file 1: Figure S2A and S2B). Consistent with these observations, colony formation was reduced in cells treated with 10, 20 and 30 μM AKBA compared to control (Fig. 2c; Additional file 1: Figure S2C). These in vitro results suggest that AKBA could significantly block the metastatic biological functions of glioblastoma cells.

### AKBA induced apoptosis of glioblastoma cells

**Fig. 3:**
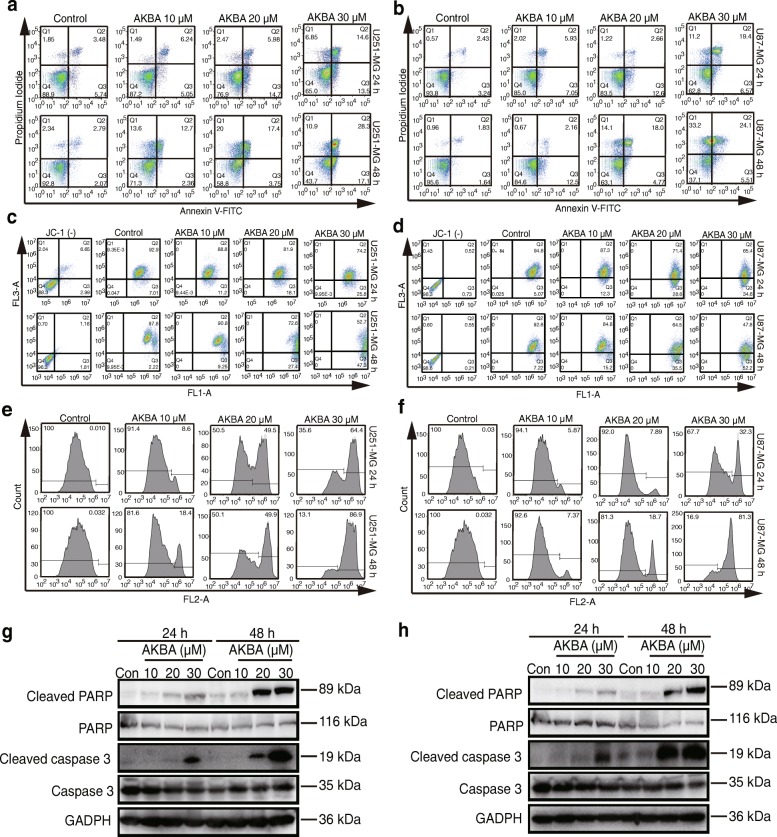
AKBA induces mitochondria-dependent apoptosis in U251 and U87-MG cells in a dose-dependent manner. Flow cytometry using Annexin V-FITC staining shows that AKBA increases apoptosis of U251 (**a**) and U87-MG (**b**) cells. Flow cytometry using JC-1 staining shows that AKBA reduces mitochondrial membrane potential in U251(**c**) and U87-MG (**d**) cells. Flow cytometry using caspase 3/7 live-cell staining shows that AKBA increases the activity of caspase 3/7 in U251 (**e**) and U87-MG (**f**) cells. Western blotting results show that AKBA induces expression of cleaved-caspase 3 and cleaved-PARP in U251 (**g**) and U87-MG (**h**) cells. The experiments were performed in triplicate

Results of apoptosis assays and membrane potential measurements showed a dose- and time-dependent increase in the percentage of early apoptotic, late apoptotic, and necrotic cells after AKBA treatment (Fig. 3a and b; Additional file 1: Figure S3A and S3B) accompanied by significant reduction in the mitochondrial membrane potential (Fig. 3c and d; Additional file 1: Figure S3C and S3D).

To explore the mechanism of AKBA-induced apoptosis, the activity of caspase-3/7 was measured and the cleavage of caspase 3 and PARP was analyzed. As shown in Fig. 3e, f, Additional file 1: Figure S3E and S3F, AKBA increased caspase-3/7 activity, especially at 30 μM where it was increased from 0.032 to 86.9% in U251 cells at 48 h. Western blotting results showed that cleavage of caspase-3 and PARP increased after exposure of U251 and U87-MG cells to 30 μM AKBA for 24 h and 20 μM or 30 μM AKBA for 48 h (Fig. 3g and h; Additional file 1: Figure S3G-J). In summary, our data are consistent with the hypothesis that the inhibition of cell growth and proliferation by AKBA is due to activation of caspase leading to apoptosis.

### Differential gene expression and enrichment analysis in U251 cells treated with AKBA

**Fig. 4:**
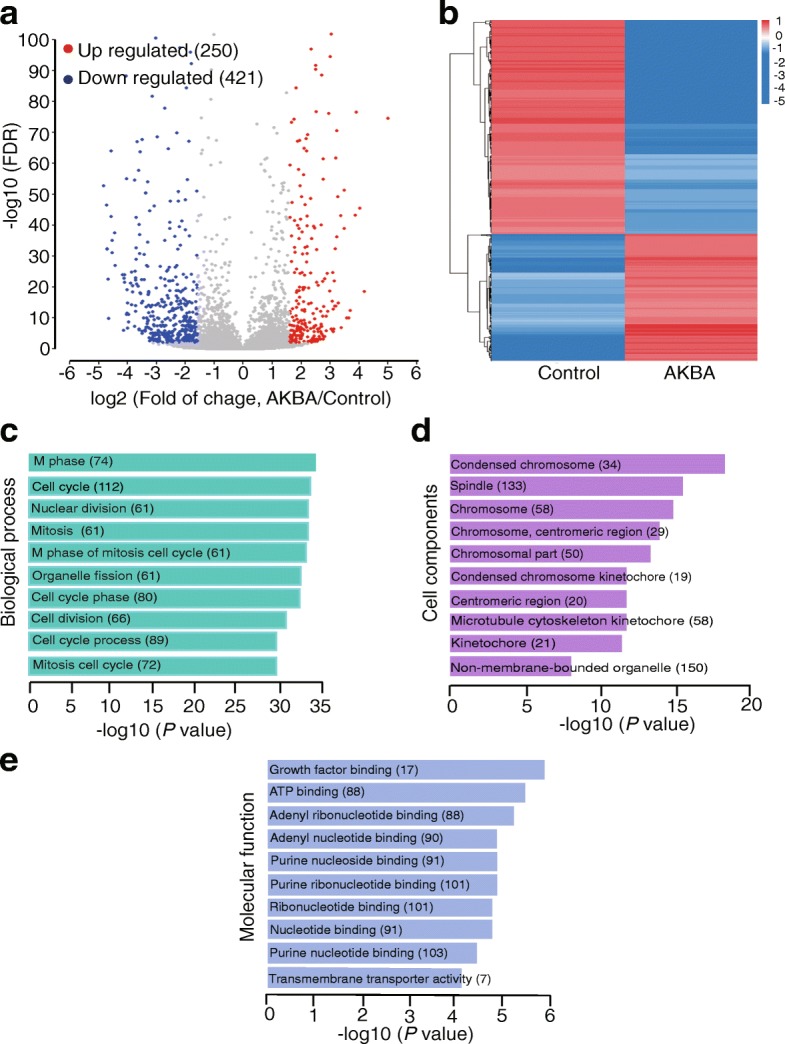
Gene ontology analysis of differentially expressed genes in U251 cells treated with AKBA for 48 h compared to control cells. **a** Volcano plot of differential expression results (up-regulated genes are in red; down-regulated genes are in blue; fold change > 3, FDR < 0.01). **b** Heatmap of differentially expressed genes. **c** Top ten enriched biological processes. **d** Top ten enriched cell components. **e** Top ten enriched molecular functions. (count ≥3, fold of change ≥3, EASE score < 0.01)

**Fig. 5:**
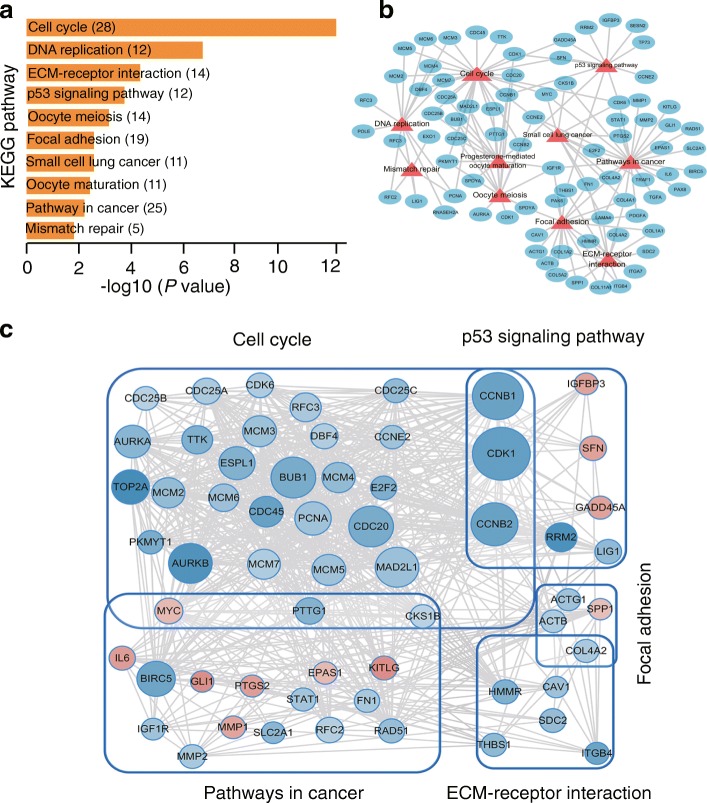
Enrichment of KEGG pathways and protein-protein interactions. **a** KEGG pathway enrichment of differentially expressed genes (fold change ≥3 and FDR < 0.01). **b** Network of KEGG pathways. **c** Gene-gene interactions. The network was visualized using Cytoscape 3.6.0. Colors of the inside nodes indicate the expression levels of quantified mRNAs (up-regulated genes are in red; down-regulated genes are in blue). Size of the nodes indicates the edge counts of the gene. The gray density of the edges defines the gene-gene interaction score identified in the STRING public database

To further explore the antitumor mechanism of AKBA, differentially expressed genes were identified by extracting and sequencing RNA from U251 and control cells incubated with 20 μM AKBA for 48 h. Compared to control cells, there were 250 up-regulated genes and 421 down-regulated genes in cells treated with AKBA (fold change > 3, FDR < 0.01, Fig. 4a and b). Gene ontology enrichment analyses of these differentially expressed genes showed that the enriched biological processes involved M phase, cell cycle, nuclear division, mitosis, and M phase of mitotic cell cycle (Fig. 4c). Affected cell components included condensed chromosomes, spindles, centromeric regions of chromosomes, chromosomal parts, and condensed chromosome kinetochores (Fig. 4d). Molecular functions included binding of growth factors, ATP, adenyl nucleotides and ribonucleotides, and purine nucleosides (Fig. 4e). KEGG pathway enrichment analysis showed enhanced cell cycle, DNA replication, ECM-receptor interaction, p53 signaling pathway, and oocyte meiosis (Fig. 5a-c). A complete list of KEGG pathways is provided in Additional file 1: Table S2.

### Validation of RNA-Seq results

To validate the results from RNA-Seq, ten differentially expressed genes were selected for qRT-PCR analysis using GAPDH as the internal reference gene. Among these genes, two were verified to be up-regulated in AKBA-treated cells whereas the other eight were identified as down-regulated. Eight genes (FOXM1, AURKA, PLK1, CDC25C, CCNB1, CDK1, AURKB and TOP2A) are involved in the cell cycle and two genes (CDKN1A, GADD45A) are associated with the p53 signaling pathway (Additional file 1: Figure S4 and S5).

### AKBA arrests cell cycle at G2/M checkpoint by regulating the p21/FOXM1/cyclin B1 signaling pathway

**Fig. 6:**
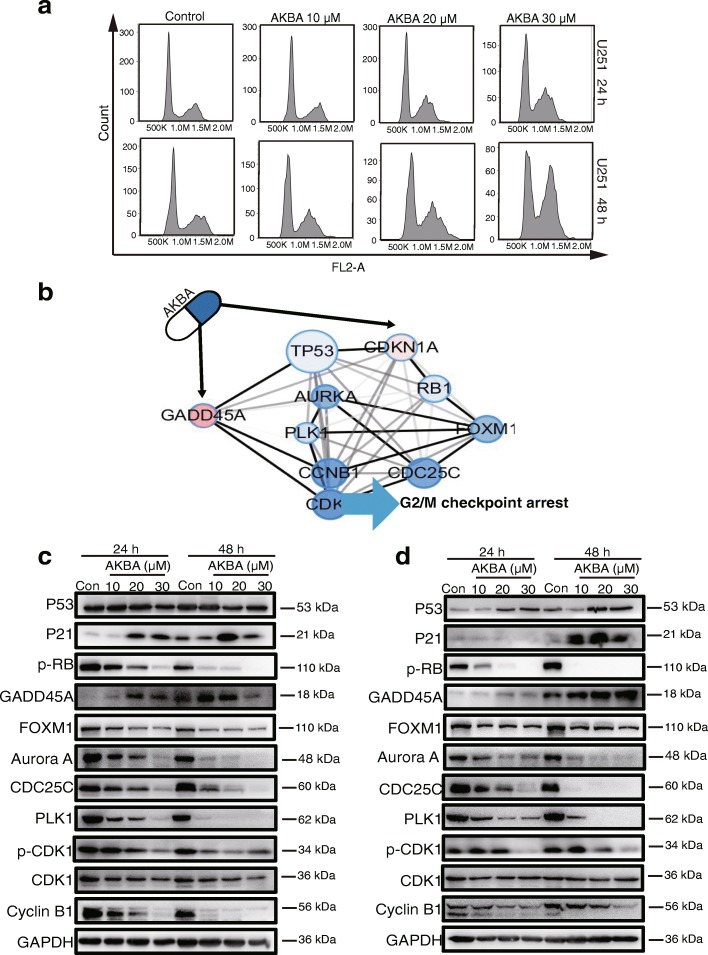
AKBA arrests cell cycle at G2/M checkpoint by regulating p21/FOXM1/cyclin B1 pathway. **a** Flow cytometry shows that AKBA arrests the cell cycle at G2/M phase in U251 cells. **b** Cytoscape visualization of the G2/M checkpoint regulatory network. **c** Protein levels of hub genes in the G2/M checkpoint of U251 cells. **d** Proteins levels of hub genes in the G2/M checkpoint of U87-MG cells. The experiments were performed in triplicate

To investigate the underlying mechanism of AKBA-induced cell cycle arrest we performed cell cycle assays and found that arrest occurred at the G2/M phase in cells incubated for 24 h or 48 h with AKBA (Fig. 6a). In addition, we checked the protein expression levels of hub genes involved in the G2/M cell cycle checkpoint (Fig. 6b). As shown in Fig. 6c, d, and Additional file 1: Figures. S6 and S7, AKBA reduced p-CDK1 and cyclin B1 protein levels in a dose- and time-dependent manner, suggesting that AKBA acts by suppressing the CDK1/cyclin B1 complex. In addition, the expression of kinases such as PLK1, Aurora A, and CDC25C responsible for the activity of the CDK1/cyclin B1 complex were also decreased by AKBA. The expression of FOXM1, a regulator of many G2/M-specific genes such as cyclin B1, Aurora A and PLK1 was also reduced after AKBA treatment. The expression of p21 was increased while that of p-RB was decreased by AKBA. However, the expression of p53 was only increased in U87-MG cells, suggesting that cell cycle arrest by AKBA was independent of p53. Finally, GADD45A, a protein that suppresses the activity of the CDK1/cyclin B1 complex through a p53-dependent/independent pathway was increased in U251 and U87-MG cells incubated with AKBA. Taken together, our results indicate that AKBA arrests the cell cycle at G2/M by regulating the p21/FOXM1/cyclin B1 and GADD45A/CDK1/cyclin B1 signaling pathways.

### AKBA arrested the cell cycle at mitosis by regulating aurora B/TOP2A signaling pathway

**Fig. 7:**
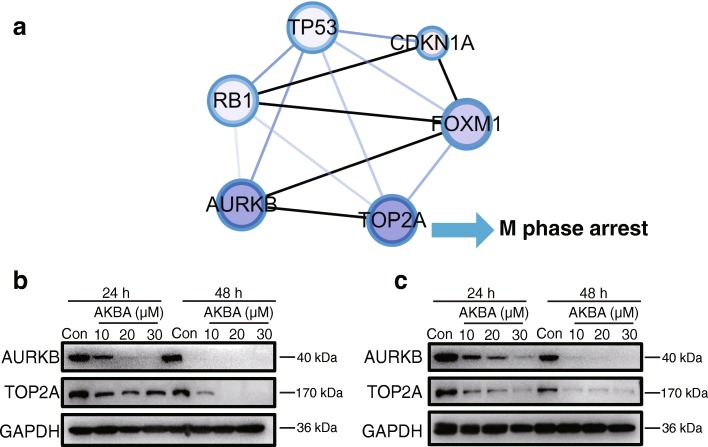
AKBA inhibits mitosis by suppressing Aurora B/TOP2A pathway. **a** Cytoscape visualization of the mitosis regulatory network. **b** Expression of Aurora B and TOP2A in U251 cells and **c** in U87-MG cells. The experiments were performed in triplicate

To determine if genes responsible for mitosis were also suppressed by AKBA, we measured the expression of Aurora B and TOP2A. AKBA down-regulated Aurora B and TOP2A in a dose- and time-dependent manner confirming that AKBA could also inhibit the mitosis of glioblastoma cells (Fig. 7a-c; Additional file 1: Figures. S8 and S9).

### AKBA suppressed the tumorigenicity of U87-MG cells in vivo

**Fig. 8:**
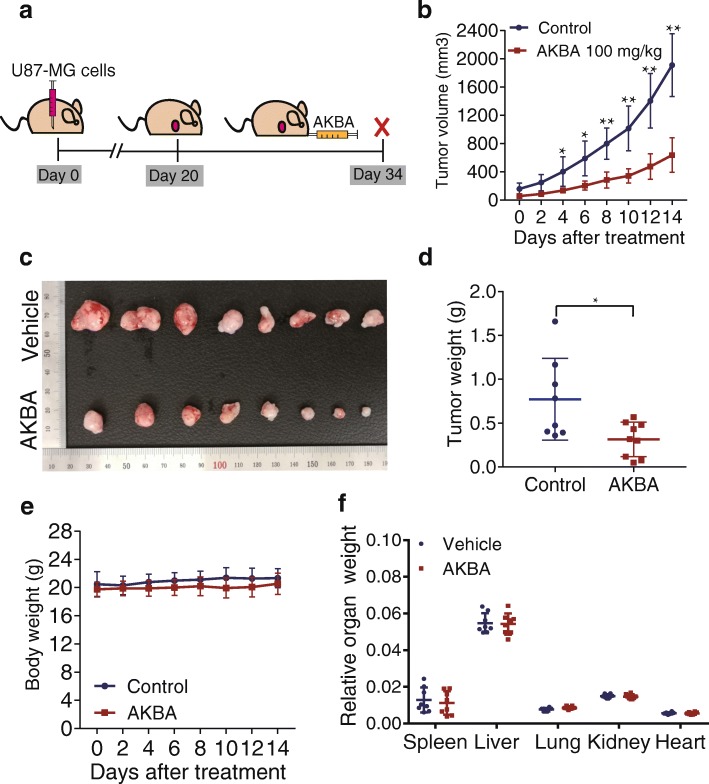
AKBA suppresses growth of xenograft tumors. **a** Schematic of xenograft tumor experiment for the investigation of anti-tumor effects of AKBA in vivo*.* **b** Changes in tumor volumes during the AKBA administration period. **c** Image of xenograft tumors. **d** Tumor weights at the end of the experiment. **e** Changes in body weight during the AKBA administration period. **f** Relative organ weights at the end of the experiment. The data were presented as mean ± SD, * *P* < 0.05, ** *P* < 0.01 vs. vehicle group

To test the antitumor activity of AKBA against glioblastoma cells in vivo, we established a xenograft model by inoculating nude mice with U87-MG cells and treating them with 100 mg/kg AKBA per day for 14 days (Fig. 8a). Oral administration of AKBA significantly decreased the volume and weight of subcutaneous U87-MG xenograft tumors in nude mice compared with vehicle controls (Fig. 8b, c and d). During the 14 days’ administration of AKBA, no obvious weight loss or abnormal behavior was detected (Fig. 8e). Furthermore, no mortality or significant changes in the colors and textures of vital organs, including the liver, kidney, brain, heart, lung, spleen, and thymus, and no significant differences in the relative organ weights was observed in the AKBA group compared to vehicle controls (Fig. 8f). These results confirmed that AKBA at a dose of 100 mg/kg did not cause obvious systemic toxicity in vivo.

**Fig. 9:**
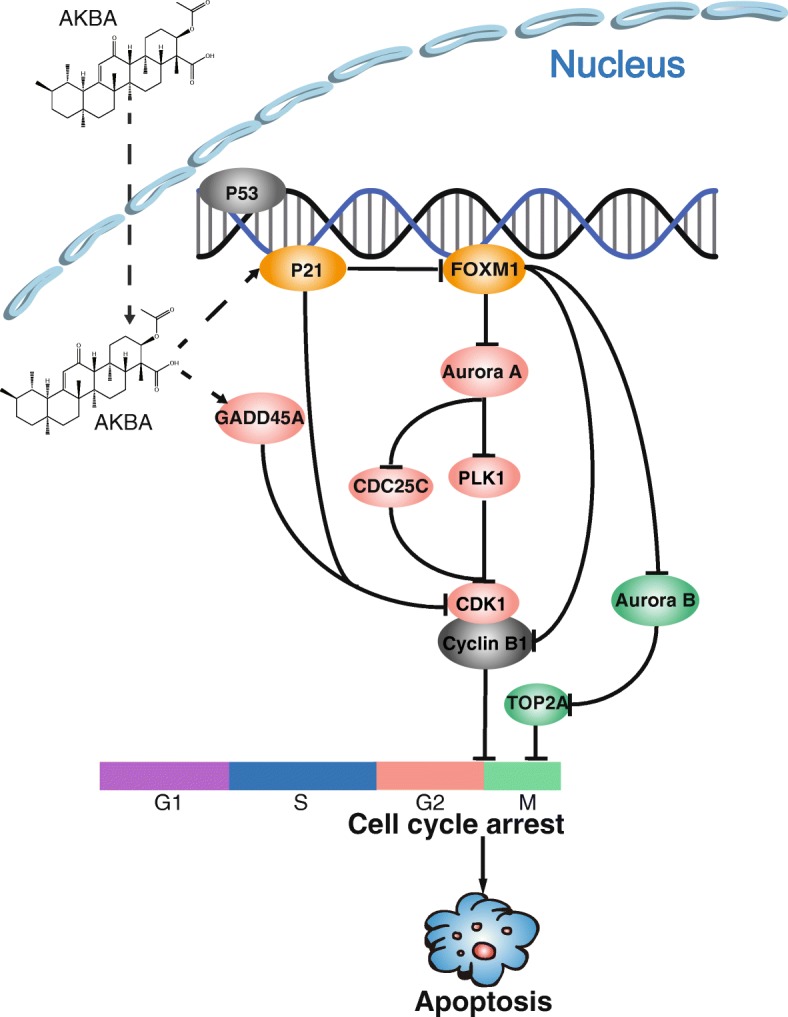
Proposed mechanistic scheme: AKBA suppresses glioblastoma by arresting cell cycle at G2/M phase. AKBA activates p21 which decreases expression of FOXM1 and its downstream target genes including Aurora A, Aurora B, PLK1, and cyclin B1. Inhibiting Aurora A suppresses the expression of PLK1 which leads to the inactivation of CDK1/cyclin B1 complex and arrests the cell cycle at the G2/M checkpoint. Inhibiting Aurora B decreases expression of TOP2A and inhibits mitosis

## Discussion

In this study, we investigated the anti-tumor activity of AKBA in U251 and U87-MG human glioblastoma cells and explored the underlying molecular mechanisms using RNA-sequencing analysis and molecular characterization of signaling pathway regulation at both mRNA and protein levels. Our results showed that AKBA inhibited cell proliferation, induced LDH release, decreased DNA synthesis, induced cell apoptosis, and inhibited the migration, invasion, and colony formation of U251 and U87-MG cells. AKBA also increased the apoptotic rate by decreasing the mitochondrial membrane potential, inhibiting the activity of caspase 3/7, and increasing expression of cleaved caspase 3 and PARP. RNA sequencing revealed that AKBA suppressed the expression of pRB, FOXM1, Aurora A, PLK1, CDC25C, CDK1, cyclin B1, Aurora B, and TOP2A while increasing expression of p21 and GADD45A, which was validated by western blotting. Our findings suggest that AKBA can arrest cell cycling at the G2/M point by regulating the p21/FOXM1/cyclin B1 pathway and that AKBA can inhibit mitosis of glioblastoma cells by down-regulating the Aurora B/ TOP2A pathway.

Apoptosis, also known as programmed cell death, is triggered by many commonly used chemopreventive agents [35]. There are two major signaling pathways controlling apoptosis, the extrinsic apoptosis pathway (death receptor) and the intrinsic apoptosis pathway (mitochondrial-dependent) [36]. As demonstrated by the assessment of mitochondrial membrane potential, AKBA caused depolarization of mitochondrial membranes in both U251 and U87-MG cells. Caspase-3/7 is considered to be the executioner caspase of mitochondria-dependent apoptosis pathways and its activation triggers PARP, which eventually leads to apoptosis [37]. In previous studies on colon cancer and hepatoma cells, AKBA induced apoptosis by increasing the expression of cleaved-caspase 3 and PARP [29, 38]. In agreement, we saw that the activity of caspase-3/7 was increased by AKBA treatment suggesting that AKBA may induce apoptosis via a mitochondria-dependent pathway.

RNA-Seq analysis has been widely used to identify novel biomarkers and mechanisms in cancer [39, 40]. We used whole transcriptome analysis to identify genes differentially expressed in cells treated in vitro with AKBA compared to control cells, and to characterize the potential biological pathways targeted by AKBA. Our RNA-Seq results showed that the cell cycle pathway was the most likely one targeted by AKBA. In eukaryotes, the cell cycle has four phases--G0/G1, S, G2, and M--which are controlled by a complex series of signaling pathways, checkpoints, kinases and other proteins. In cancer cells, as a result of genetic mutations or defective upstream signaling pathways, the cell cycle is dysregulated resulting in uncontrolled cell proliferation, growth, invasion, and metastasis [41]. At the G2/M checkpoint, the CDK1/cyclin B1 complex is crucial in regulating the G2/M phase transition. Previous studies showed that BA145, an analogue of boswellic acid, inhibited the proliferation of pancreatic cancer cells by arresting them at the G2/M phase, which is associated with decreased expression of cyclin B and CDK-1. Treatment of glioblastoma cells with AKBA significantly decreased the ratio of p-CKD1/CDK1 and cyclin B1 suggesting that AKBA arrested the cell cycle at the G2/M phase, thereby inhibiting cell proliferation.

Although the CDK1/cyclin B1 complex can be auto-activated, PLK1 is generally believed responsible for the initial activation of CDK1/cyclin B1 by localizing cyclin B1 and directly regulating the complex. PLK1 phosphorylates cyclin B in centrosomes leading to nuclear accumulation of p-cyclin B1 during prophase. PLK1 can also regulate CDK1/cyclin B1 by the CDC25C/WEE1/MYT1 axis. PLK1 promotes the nuclear localization of CDC25C by phosphorylating its Ser198 during prophase [42, 43]. The activity of PLK1 is also regulated by Aurora A, a kinase whose overexpression inactivates the DNA damage checkpoint during G2. In this study, the protein levels of Aurora A, PLK1, and CDC25C were decreased by AKBA suggesting that it reduced the activity of the CDK1/cyclin B1 complex through the Aurora A/PLK1 and Aurora A/PLK1/CDC25C axes.

FOXM1 is a transcription factor in the forkhead box family [44] that is essential for chromosomal stability and regulates the expression of many G2/M-specific genes, such as CCNB1, PLK1, AURKB, and centromere protein F [45, 46]. In this study, AKBA reduced the expression of FOXM1 and its target genes, cyclin B1, PLK1, AURKA and AURKB, which may mean that the AKBA-mediated G2/M phase arrest is caused in part by the down-regulation of FOXM1. Both Aurora A and FOXM1 are regulated by the p53 signaling pathway, which was enriched by differential gene expression in AKBA-treated cells. Through transcriptional induction of p21/waf1, p53 can also suppress CDK, which in turn leads to the formation of repressive Rb/E2F complexes. The FOXM1 gene is a downstream target of E2F [47] and E2F expression was reduced by AKBA. In addition to inhibiting CDK1 via p21/waf1, p53 can also suppress the kinase activity by upregulating CDK1’s downstream target, GADD45A [48]. In previous studies, boswellic acid inhibited cell proliferation via a p21-dependent signal pathway, independent of p53 [27, 49]. In this study, the expression of p21, pRB, and GADD45A increased in U251 and U87-MG cells after incubation with AKBA. Consistent with previous reports, AKBA only increased the expression of p53 in U87-MG cells and not in U251 cells. We confirmed that AKBA exerts its anti-tumor effects through a p53-independent pathway and arrested the cell cycle of glioblastoma cells at the G2/M phase.

In addition to arresting the cell cycle at G2/M, AKBA also exerted anti-tumor effects by suppressing the activity of some kinases at mitosis. Aurora B is a regulator of the kinetochore-microtubule attachment [50] to ensure accurate chromosome segregation, alignment, and cytokinesis [51]. Aurora B, overexpressed in many cancer cells, might play an important role in tumorigenesis and could be a potential target for cancer diagnosis and therapy [52]. We found that AKBA suppressed the expression of Aurora B and, therefore, that it might be a potential anticancer target for AKBA.TOP2A encodes a DNA topoisomerase II which regulates the topologic state of DNA in the process of transcription [53]. Previous studies showed that acetyl-boswellic acids were novel catalytic inhibitors of human topoisomerases I and II alpha [54] and we confirmed here that the expression of TOP2A was also suppressed by AKBA in U251 and U87-MG cells. TOP2A prevents incorrect microtubule-kinetochore attachments and is regulated by the activation of Aurora B kinase [55]. Thus, our results are consistent with the idea that the anti-proliferative effects of AKBA are mediated through the Aurora B/TOP2A signaling pathway.

Using a mouse xenograft glioblastoma tumor model, we found that oral administration of 100 mg/kg AKBA suppressed the tumorigenicity of U87-MG cells in vivo*.* AKBA is highly lipophilic which facilitates its crossing the blood-brain barrier [56, 57]. In addition, *Boswellia serrata* extracts, as well as KBA and AKBA, are not P-glycoprotein (Pgp) substrates and are identified as potent inhibitors of Pgp in porcine brain capillary endothelial cells and human lymphocytic leukemia parenteral cell lines [58, 59]. These characteristics could also facilitate AKBA’s brain penetration and enhance its anti-glioblastoma effects in vivo.

## Conclusion

In conclusion, we found that AKBA exerted anti-tumor effects against human glioblastoma by inducing mitochondrial-dependent apoptosis via cell cycle arrest at the G2/M phase. The mechanism involved regulation of the p21/FOXM1/cyclin B1 signaling pathway and suppression of mitosis by downregulation of the Aurora B/TOP2A pathway (Fig. 9). This body of evidence supports the potential of AKBA as a promising chemotherapy drug in the treatment of glioblastoma.

## Additional file

Additional file 1: Supplementary figures and tables. (DOCX 1941 kb)

## Abbreviations

AKBA: 3-O-Acetyl-11-keto-β-boswellic acid
AURKB: Aurora kinase B
CCNB1: Cyclin B1
CENPF: Centromere protein F
DMEM: Dulbecco’s Modified Eagle’s Medium
GBM: Glioblastoma
JC-1: 5,5′,6,6′-tetrachloro-1,1′,3,3′-tetraethylbenzimidazolocarbocyanine iodide
LDH: Lactate dehydrogenase
PLK1: polo-like kinase 1
TMZ: Temozolomide
