# Correction: 3-O-acetyl-11-keto-β-boswellic acid exerts anti-tumor effects in glioblastoma by arresting cell cycle at G2/M phase

**DOI:** 10.1186/s13046-022-02454-7

**Published:** 2022-08-03

**Authors:** Wan Li, Jinyi Liu, Weiqi Fu, Xiangjin Zheng, Liwen Ren, Shiwei Liu, Jinhua Wang, Tengfei Ji, Guanhua Du

**Affiliations:** 1 The State Key Laboratory of Bioactive Substance and Function of Natural Medicines, Beijing, 100050 China; 2 Key Laboratory of Drug Target Research and Drug Screen, Institute of Materia Medica, Chinese Academy of Medical Science and Peking Union Medical College, Beijing, 100050 China; 3 Ethnic Drug Screening & Pharmacology Center, Key Laboratory of Chemistry in Ethnic Medicinal Resources, State Ethnic Affairs Commission & Ministry of Education, Yunnan Minzu University, Kunming, 650500 China; 4 Department of Endocrinology, Shanxi DAYI Hospital, Shanxi Medical University, Taiyuan, 030002 Shanxi China

**Correction: J Exp Clin Cancer Res 37, 132 (2018)**

**https://doi.org/10.1186/s13046-018-0805-4**

Following publication of the original article [1], an error was identified in Fig. 3; specifically:

- Figure 3e and 3f, panels “U251-MG 48h Control” and “U87-MG 48h Control” were inadvertently duplicated

**Fig. 3:**
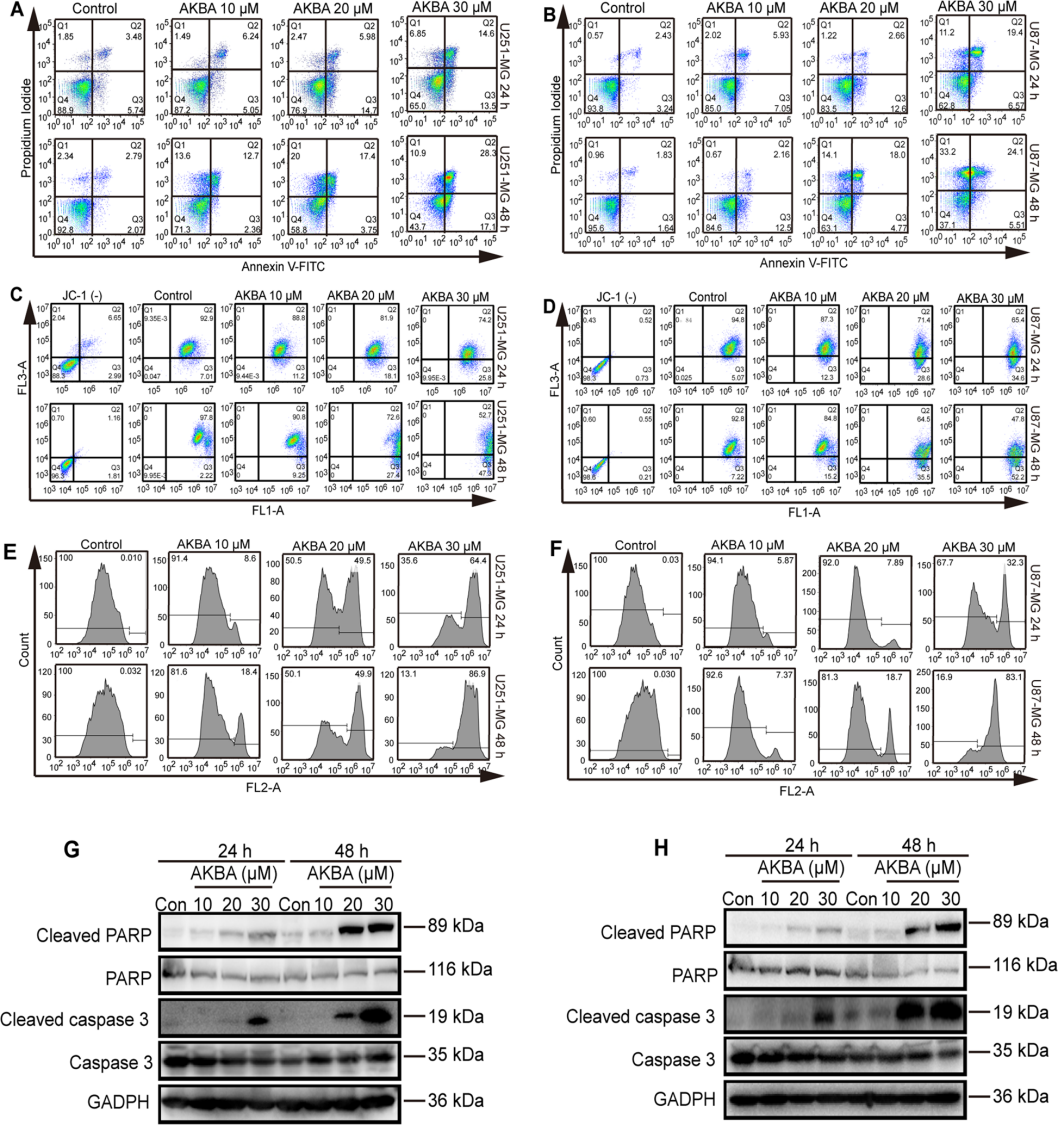
AKBA induces mitochondria-dependent apoptosis in U251 and U87-MG cells in a dose-dependent manner. Flow cytometry using Annexin V-FITC staining shows that AKBA increases apoptosis of U251 (**a**) and U87-MG (**b**) cells. Flow cytometry using JC-1 staining shows that AKBA reduces mitochondrial membrane potential in U251(**c**) and U87-MG (**d**) cells. Flow cytometry using caspase 3/7 live-cell staining shows that AKBA increases the activity of caspase 3/7 in U251 (**e**) and U87-MG (**f**) cells. Western blotting results show that AKBA induces expression of cleaved-caspase 3 and cleaved-PARP in U251 (**g**) and U87-MG (**h**) cells. The experiments were performed in triplicate

The correction does not have any effect on the results or conclusions of the paper.
