# Supplementary material for: 3-O-acetyl-11-keto-β-boswellic acid exerts anti-tumor effects in glioblastoma by arresting cell cycle at G2/M phase
Source: J Exp Clin Cancer Res. 2018 Jul 3;37:132. doi: 10.1186/s13046-018-0805-4 (PMC6029111; doi:10.1186/s13046-018-0805-4)
Supplement: Supplementary file 1 — Supplementary figures and tables. (DOCX 1941 kb) [file 13046_2018_805_MOESM1_ESM.docx]

Supplementary data

AKBA exerts anti-tumor effects in glioblastoma by arresting cell cycle at G2/M phase

**Wan Li**^1,2,*^**, Jinyi Liu**^2,3*^**, Weiqi Fu**^1,2^ **, Xiangjin Zheng**^1,2,^**, Liwen Ren**^1,2^**, Shiwei Liu**^4^, **Jinhua Wang**^1,2,£^**, Tengfei Ji**^1,£^**, Guanhua Du**^1,2,£^

^1^The State Key Laboratory of Bioactive Substance and Function of Natural Medicines;

^2^Key Laboratory of Drug Target Research and Drug Screen, Institute of Materia Medica, Chinese Academy of Medical Science and Peking Union Medical College. Beijing, China 100050.

^3^Ethnic Drug Screening & Pharmacology Center, Key Laboratory of Chemistry in Ethnic Medicinal Resources, State Ethnic Affairs Commission & Ministry of Education, Yunnan Minzu University, Kunming, China, 650500.

^4^Department of Endocrinology, Shanxi DAYI Hospital, Shanxi Medical University, Taiyuan, Shanxi, China, 030002.

**Running title:** AKBA exerts anti-tumor effects in glioblastoma

^*^ These authors contributed equally to this work.

^£^ These authors should be considered as corresponding authors:

Jinhua Wang, Email: wjh@imm.ac.cn, Fax: 86-10-63165184

Tengfei Ji, Email: [jitf@imm.ac.cn](mailto:jitf@imm.ac.cn), Fax: 86-10-63017757

Guanhua Du, Email: dugh@imm.ac.cn, Fax: 86-10-63165184

**Table S1: Primers for differential expression genes and GAPDH**

| Gene name | Primer sequence |
| --- | --- |
| CDK1 | Forward: 5’-GGGGTCAGCTCGTTACTCAA -3’ |
|  | Reverse: 5’-CACTTCTGGCCACACTTCAT -3’ |
| CCNB1 | Forward: 5’-TGTTGGTTTCTGCTGGGTGT -3’ |
|  | Reverse: 5’-TGCCATGTTGATCTTCGCCT -3’ |
| PLK1 | Forward: 5’- CGTGACCTACATCGACGAGA-3’ |
|  | Reverse: 5’- GGAGGGCAGCTATTAGGAGG-3’ |
| ARUKA | Forward: 5’-ACAGGCATCATGGACCGATC -3’ |
|  | Reverse: 5’-AAGGAATGCGCTGGGAAGAA -3’ |
| CDC25C | Forward: 5’- ACTGAGTTGCTGAGGTGTCG-3’ |
|  | Reverse: 5’- GCCTCTTTCTGCTCAGGGTT-3’ |
| GADD45A | Forward: 5’-CTGAACGGTGATGGCATCTG -3’ |
|  | Reverse: 5’- TTGAACTCACTCAGCCCCTT-3’ |
| FOXM1 | Forward: 5’- TTCCCTGCACGACATGTTTG-3’ |
|  | Reverse: 5’- GCTCTGGATTCGGTCGTTTC-3’ |
| P21-CDKN1A | Forward: 5’- CCATCCCTCCCCAGTTCATT-3’ |
|  | Reverse: 5’-AAGACAACTACTCCCAGCCC -3’ |
| AURKB | Forward: 5’-GACACCCGACATCTTAACGC -3’ |
|  | Reverse: 5’-CGCCCTCCTTCTCTATCTGG -3’ |
| TOP2A | Forward: 5’-TGGTGGCAAGGATTCTGCTA -3’ |
|  | Reverse: 5’-CCACCCAGTACCGATTCCTT -3’ |
| GAPDH | Forward: 5’- TCCAAAATCAAGTGGGGCGA-3’ |
|  | Reverse: 5’- TGATGACCCTTTTGGCTCCC -3’ |

| **Table S2:**  **Enriched KEGG pathways upon the treatment of AKBA** | | | |
| --- | --- | --- | --- |
| **Term** | **Count** | **PValue** | **Genes** |
| hsa04110: Cell cycle | 28 | 1.38E-12 | E2F2, DBF4, PKMYT1, TTK, SFN, PTTG1, CCNE2, CDC45, MCM7, BUB1, MYC, CDK1, CDK6, ESPL1, CDC20, MCM2, MCM3, CDC25C, MCM4, MCM5, CDC25A, CDC25B, MCM6, CCNB1, CCNB2, MAD2L1, PCNA, GADD45A |
| hsa03030: DNA replication | 12 | 1.79E-07 | RFC3, MCM7, RFC2, LIG1, POLE, PCNA, MCM2, MCM3, RNASEH2A, MCM4, MCM5, MCM6 |
| hsa04512: ECM-receptor interaction | 14 | 5.33E-05 | COL4A2, COL4A1, ITGB4, COL5A2, SDC2, HMMR, LAMA4, ITGA7, COL1A2, COL1A1, THBS1, COL11A1, FN1, SPP1 |
| hsa04115: p53 signaling pathway | 12 | 1.34E-04 | CCNE2, CCNB1, CDK1, CCNB2, RRM2, CDK6, SFN, SESN2, THBS1, IGFBP3, GADD45A, TP73 |
| hsa04114: Oocyte meiosis | 14 | 8.32E-04 | CDK1, PKMYT1, AURKA, CDC20, ESPL1, PTTG1, CDC25C, SPDYA, CCNB1, CCNE2, IGF1R, CCNB2, MAD2L1, BUB1 |
| hsa04510: Focal adhesion | 19 | 0.002456 | ACTB, CAV1, COL4A2, COL4A1, PDGFA, ITGB4, COL5A2, SPDYA, PAK6, ACTG1, IGF1R, LAMA4, ITGA7, COL1A2, COL1A1, THBS1, COL11A1, FN1, SPP1 |
| hsa05222: Small cell lung cancer | 11 | 0.003112 | TRAF1, CCNE2, E2F2, CKS1B, LAMA4, COL4A2, COL4A1, PTGS2, CDK6, MYC, FN1 |
| hsa04914: Progesterone-mediated oocyte maturation | 11 | 0.003702 | SPDYA, CCNB1, CDK1, IGF1R, MAD2L1, CCNB2, BUB1, PKMYT1, CDC25C, CDC25A, CDC25B |
| hsa05200: Pathways in cancer | 25 | 0.00725 | TRAF1, E2F2, CKS1B, PTGS2, PDGFA, KITLG, MMP2, MMP1, GLI1, CCNE2, IGF1R, SLC2A1, PAX8, TGFA, MYC, FN1, COL4A2, IL6, COL4A1, EPAS1, BIRC5, CDK6, STAT1, RAD51, LAMA4 |
| hsa03430: Mismatch repair | 5 | 0.015791 | EXO1, RFC3, RFC2, LIG1, PCNA |
| hsa05130: Pathogenic Escherichia coli infection | 7 | 0.035329 | ACTG1, ACTB, ARHGEF2, OCLN, LY96, CLDN1, TUBA1A |
| hsa00260: Glycine, serine and threonine metabolism | 5 | 0.042933 | CTH, DMGDH, PSAT1, PIPOX, CBS |
| hsa05020: Prion diseases | 5 | 0.062579 | IL6, C9, C5, HSPA5, IL1A |
| hsa03410: Base excision repair | 5 | 0.062579 | HMGB1, UNG, LIG1, POLE, PCNA |


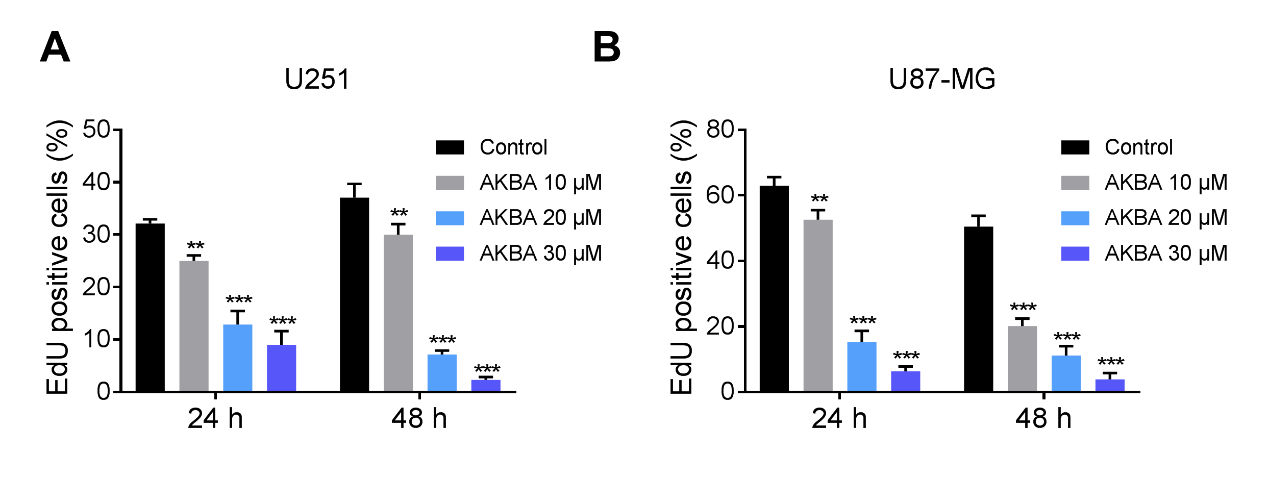


**Figure S1. Quantification of the proportion in EdU positive cells.** **(A)** The proportion of EdU positive cells after the treatment of AKBA in U251 cells. **(B)** The proportion of EdU positive cells after the treatment of AKBA in U87-MG cells. The experiments were performed in triplicate, and the data were presented as mean ± SD, ** *P* < 0.01, ****P* < 0.0001 vs. control group.

**
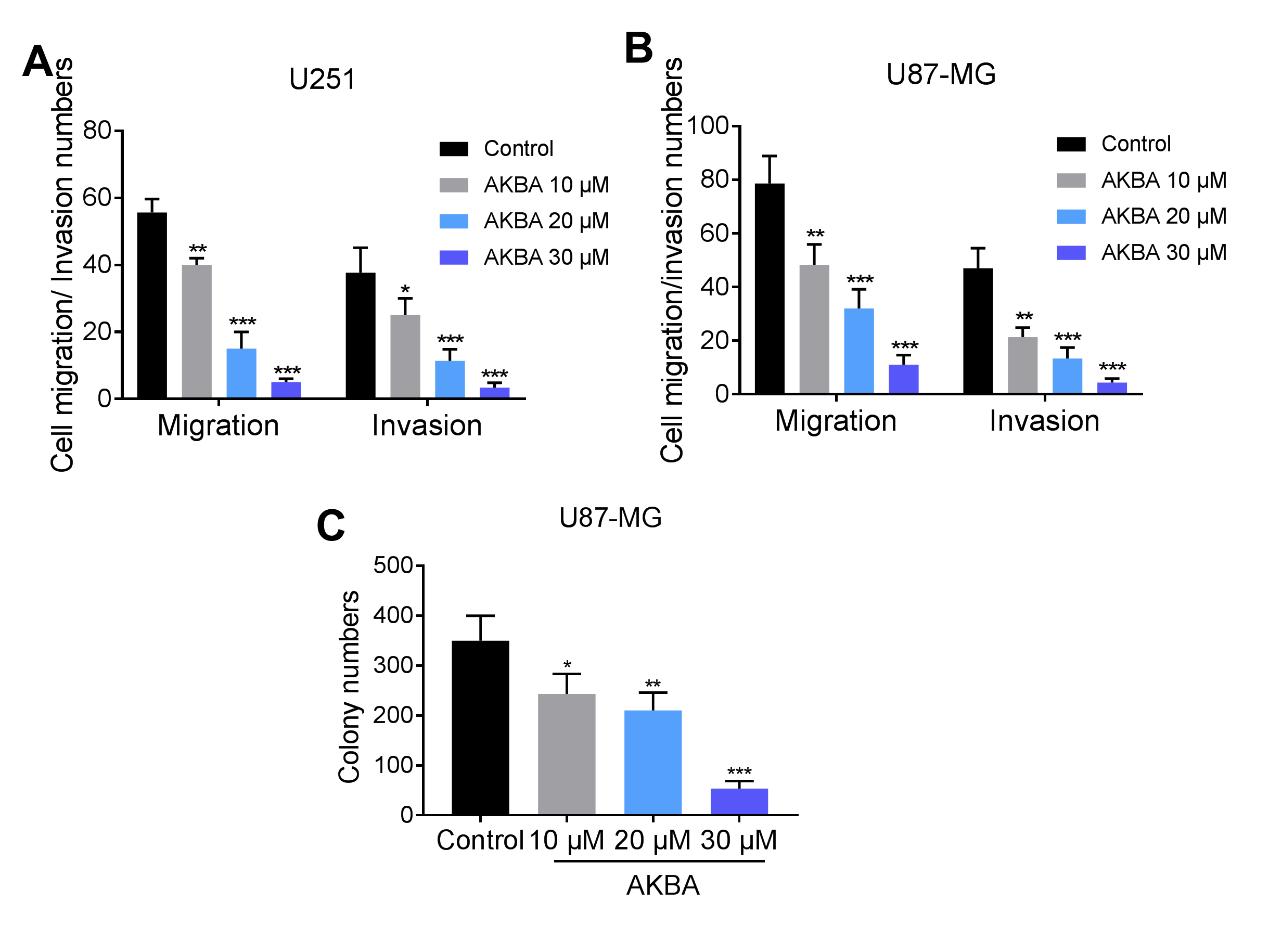
**

**Figure S2. Quantification of cell migration, cell invasion, and colony formation numbers. (A)** Cell migration and invasion numbers after the treatment of AKBA in U251 cells. **(B)** Cell migration and invasion numbers after the treatment of AKBA in U87-MG cells.in U87-MG cells. **(C)** Colony formation numbers after the treatment of AKBA in U87-MG cells. The experiments were performed in triplicate, and the data were presented as mean ± SD, * *P* < 0.05, ** *P* < 0.01, ****P* < 0.0001 vs. control group.

**
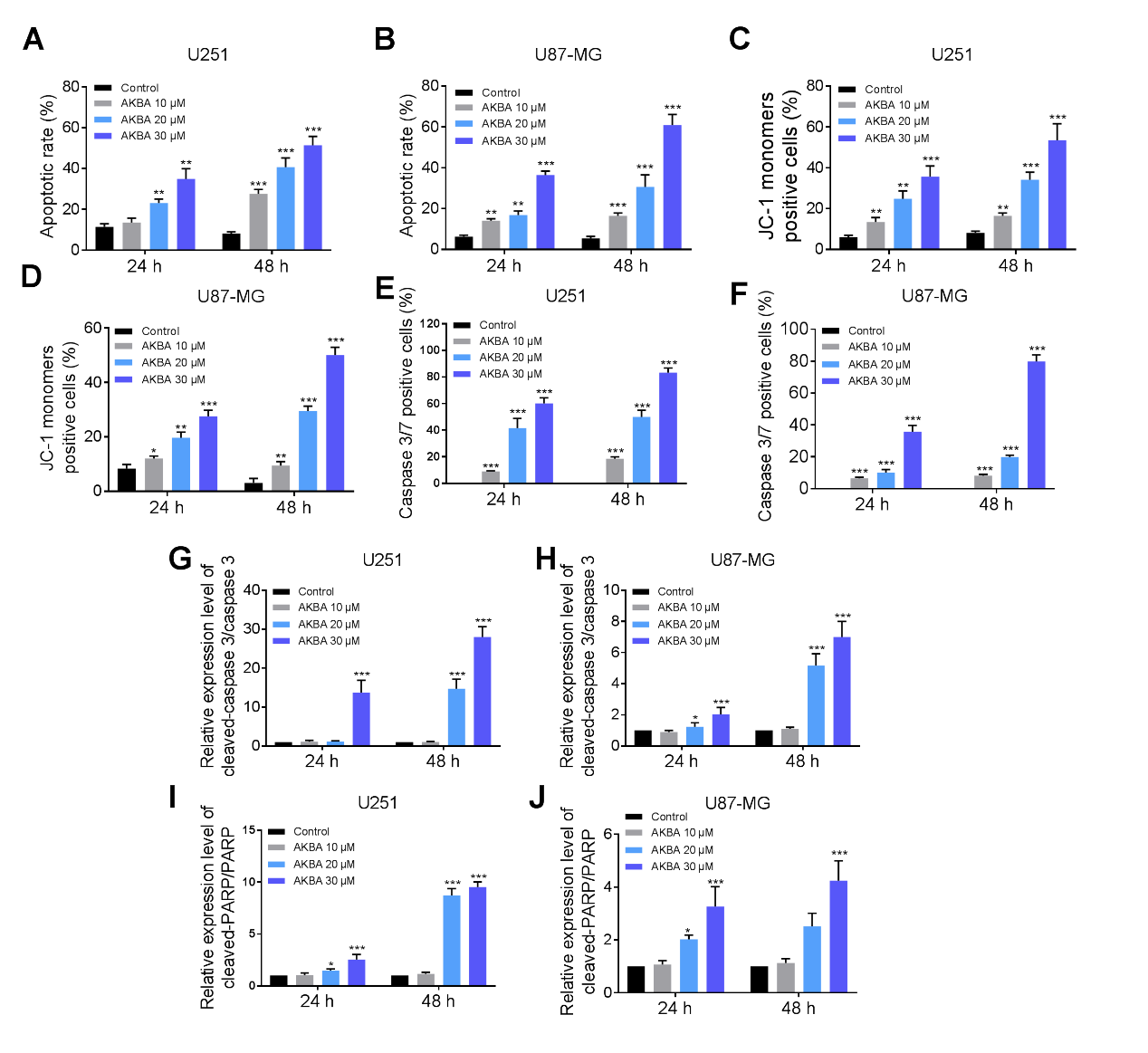
**

**Figure S3. AKBA induced mitochondrial dependent apoptosis in U251 and U87-MG cells. (A)** Quantification of the apoptotic rate of U251 cells after the treatment of AKBA. **(B)** Quantification of the apoptotic rate in U87-MG cells after the treatment of AKBA. **(C)** Quantification of the percentage of JC-1 monomers positive U251 cells after the treatment of AKBA. **(D)** Quantification of the percentage of JC-1 monomers positive U87-MG cells after the treatment of AKBA. **(E)** Quantification of the percentage of caspase 3/7 positive U251 cells after the treatment of AKBA. **(F)** Quantification of the percentage of caspase 3/7 positive U87-MG cells after the treatment of AKBA. **(G)** Quantification of the relative expression level of cleaved caspase 3/caspase 3 in U251 cells after the treatment of AKBA. **(H)** Quantification of the relative expression level of cleaved caspase 3/caspase 3 of U87-MG cells after the treatment of AKBA. **(I)** Quantification of the relative expression level of cleaved PARP/PARP of U251 cells after the treatment of AKBA. **(J)** Quantification of the relative expression level of cleaved PARP/PARP of U87-MG cells after the treatment of AKBA. The experiments were performed in triplicate, and the data were presented as mean ± SD, * *P* < 0.05, ** *P* < 0.01, ****P* < 0.0001 vs. control group.

**
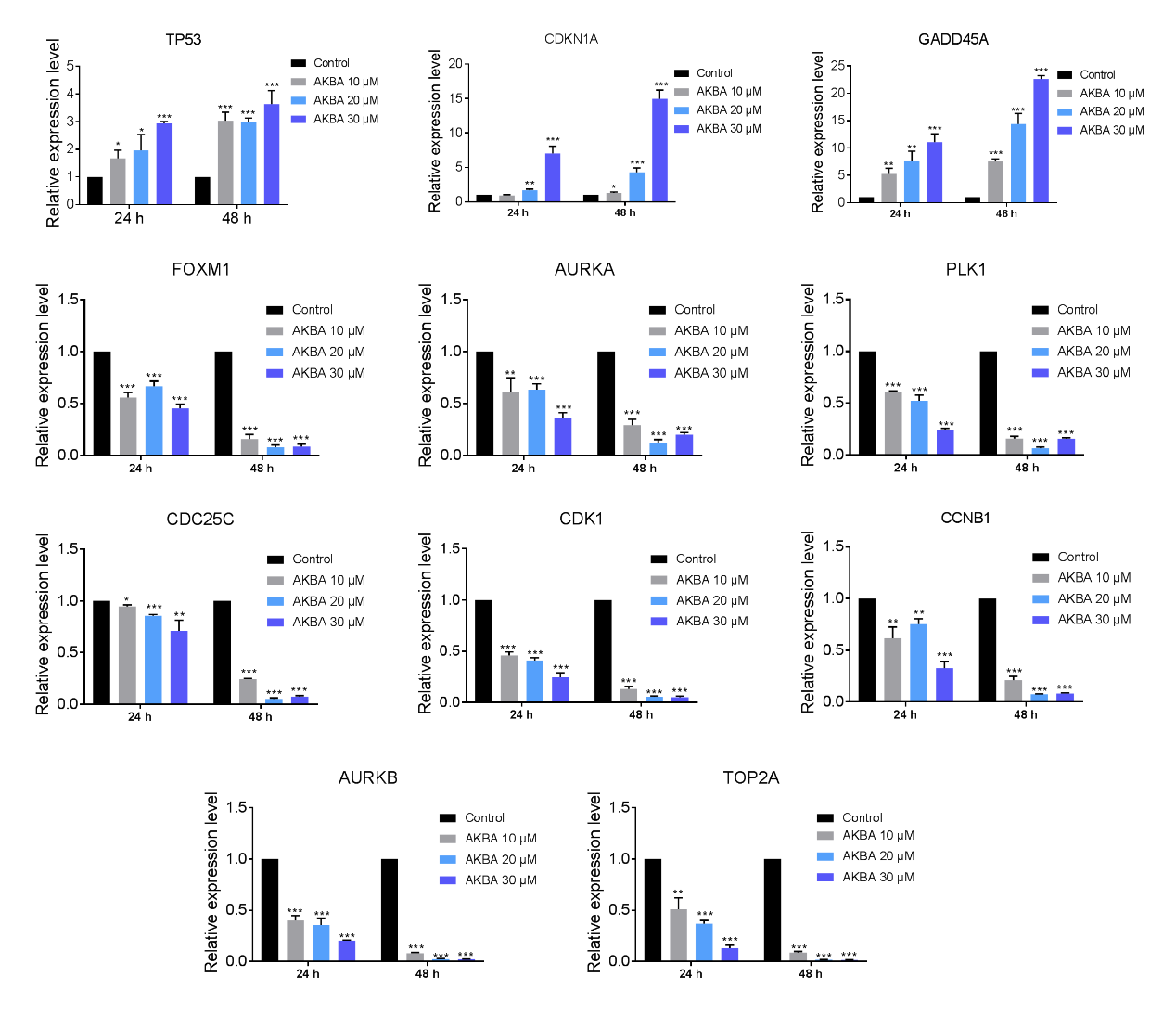
**

**Figure S4. Quantification of the mRNA expression levels of TP53, CDKN1A, GADD45A, FOXM1, AURKA, PLK1, CDC25C, CDK1, CCNB1, AURKB, and TOP2A of U251 cells after the treatment of AKBA.** The experiments were performed in triplicate, and the data were presented as mean ± SD, * *P* < 0.05, ** *P* < 0.01, ****P* < 0.0001 vs. control group.

**
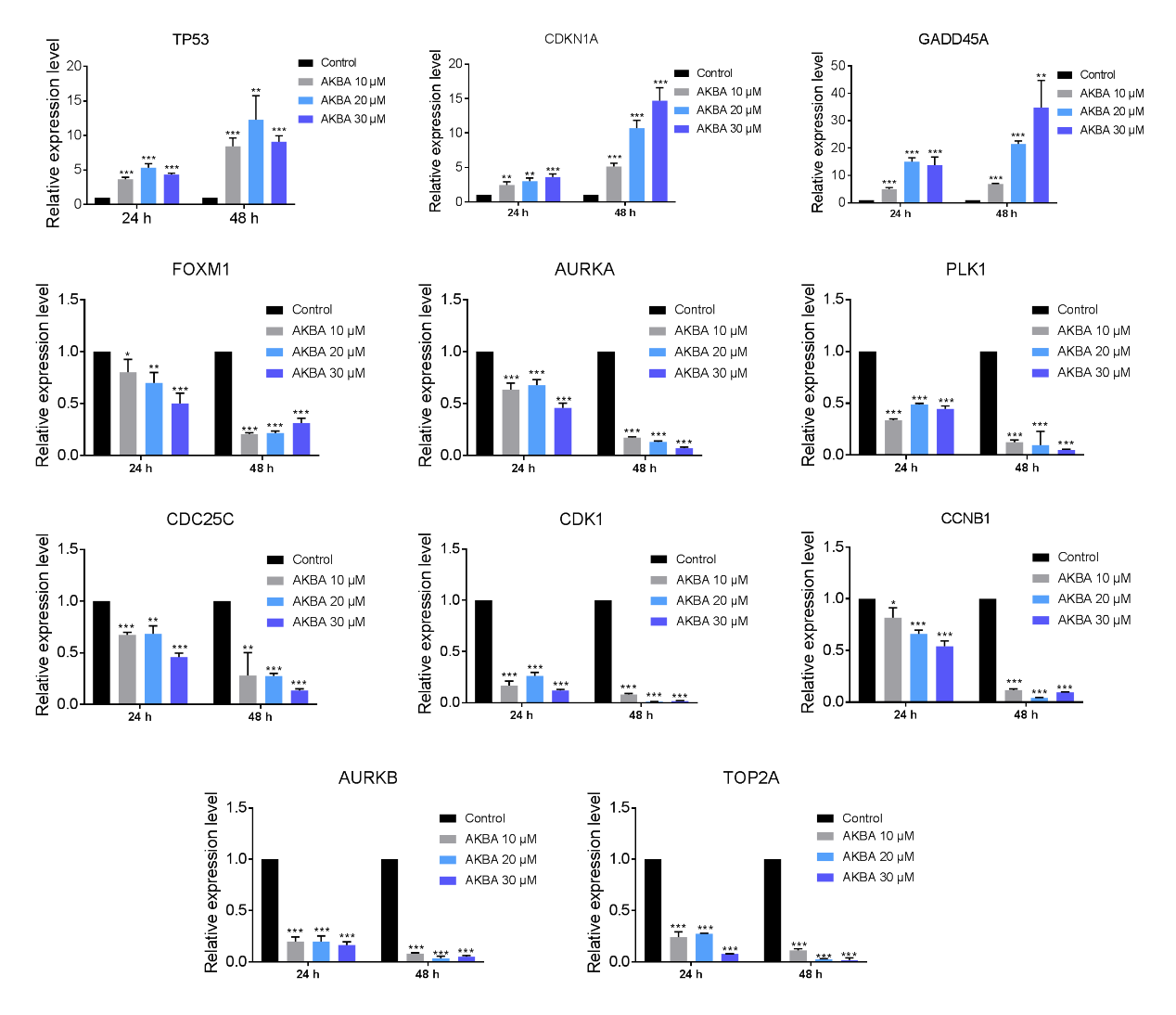
**

**Figure S5. Quantification of the mRNA expression level of TP53, CDKN1A, GADD45A, FOXM1, AURKA, PLK1, CDC25C, CDK1, CCNB1, AURKB, and TOP2A of U87-MG cells after the treatment of AKBA.** The experiments were performed in triplicate, and the data were presented as mean ± SD, * *P* < 0.05, ** *P* < 0.01, ****P* < 0.0001 vs. control group.

**
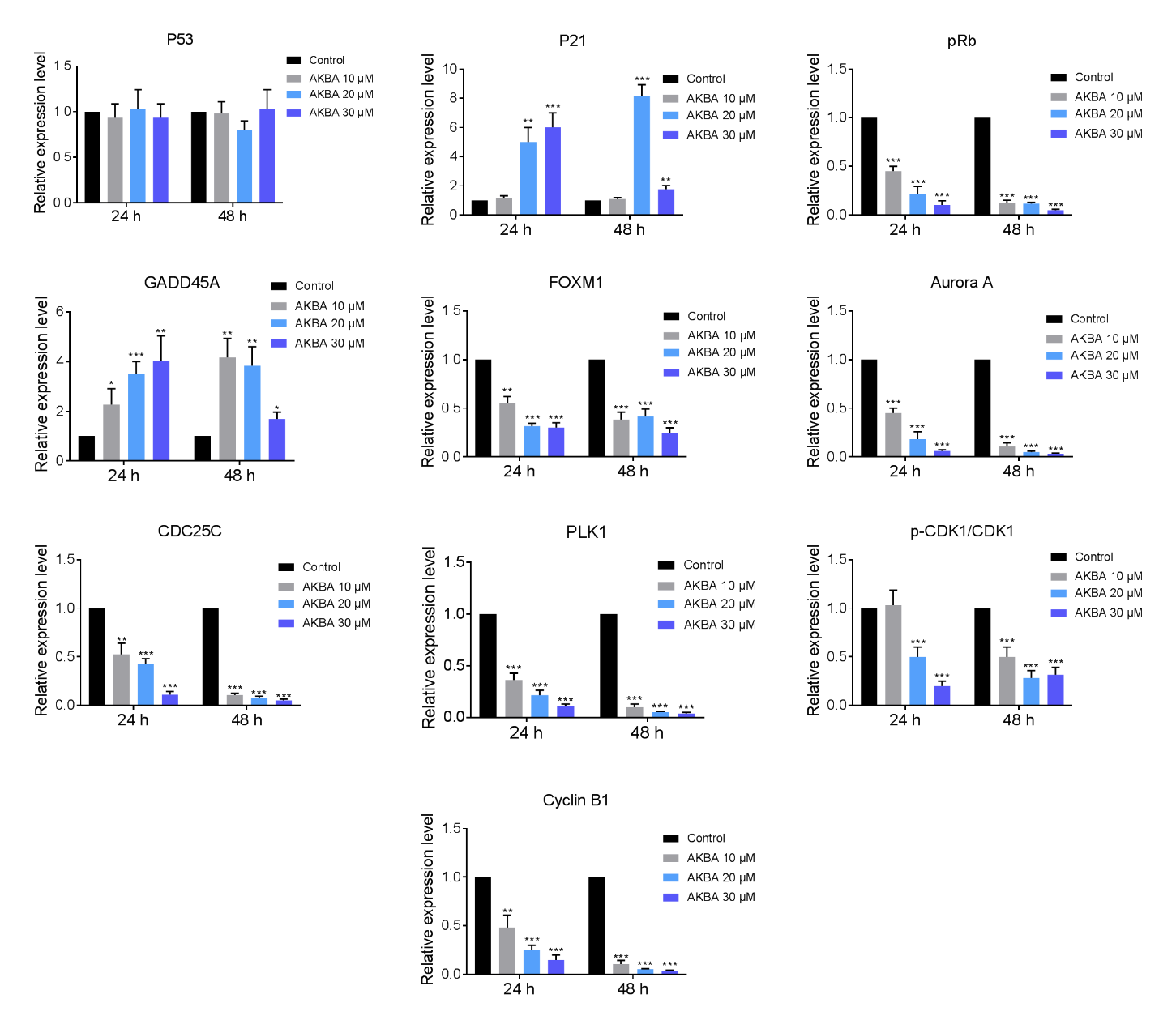
**

**Figure S6.** **Quantification of the protein expression levels of P53, P21, pRB, GADD45A, FOXM1, Aurora A, CDC25C, PLK1, p-CDK1/CDK1, and Cyclin B1 in U251 cells after the treatment of AKBA.** The experiments were performed in triplicate, and the data were presented as mean ± SD, * *P* < 0.05, ** *P* < 0.01, ****P* < 0.0001 vs. control group.

**
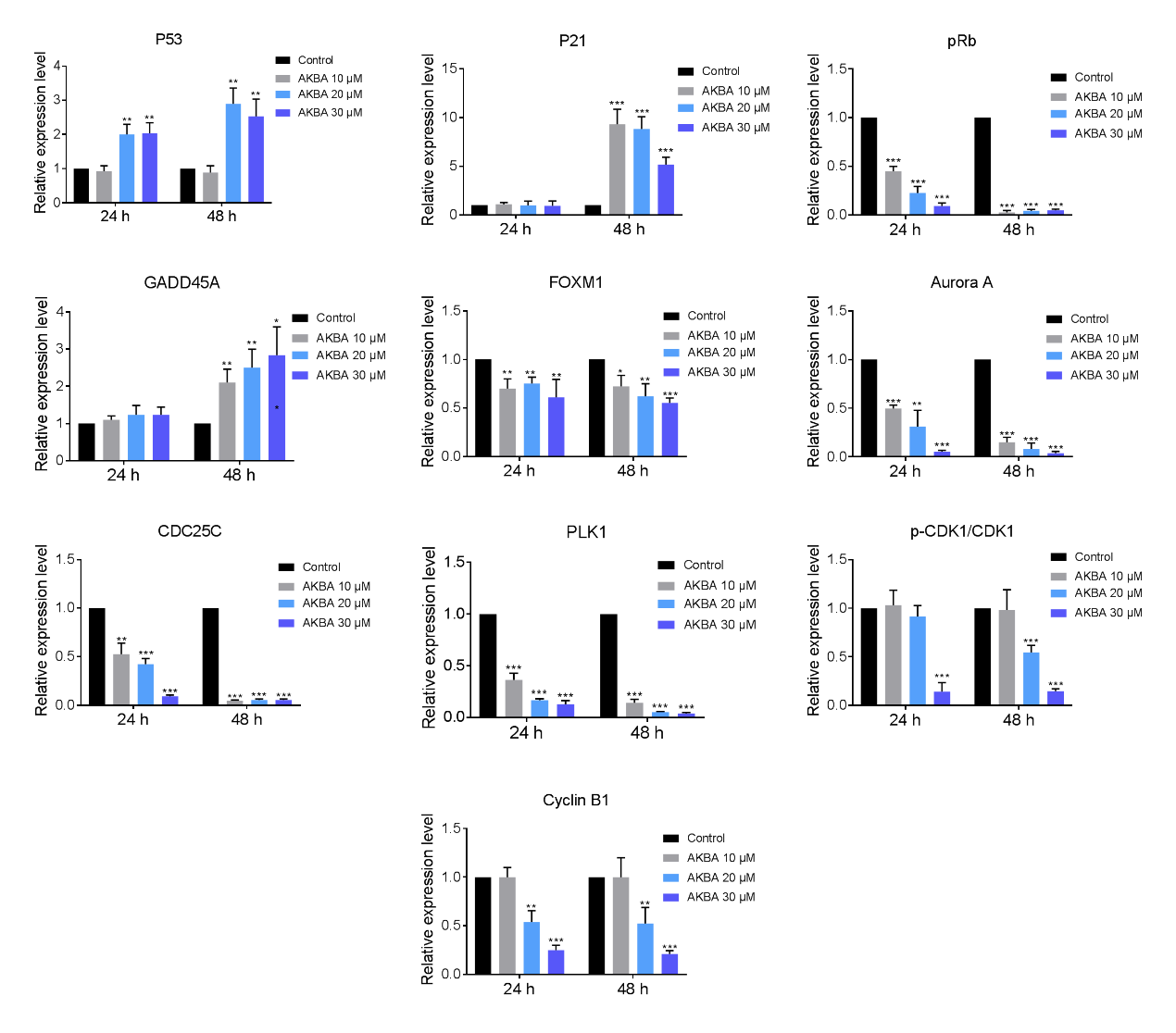
**

**Figure S7. Quantification of the protein expression levels of P53, P21, pRB, GADD45A, FOXM1, Aurora A, CDC25C, PLK1, p-CDK1/CDK1, and Cyclin B1 in U87-MG cells after the treatment of AKBA.** The experiments were performed in triplicate, and the data were presented as mean ± SD, * *P* < 0.05, ** *P* < 0.01, ****P* < 0.0001 vs. control group.

**
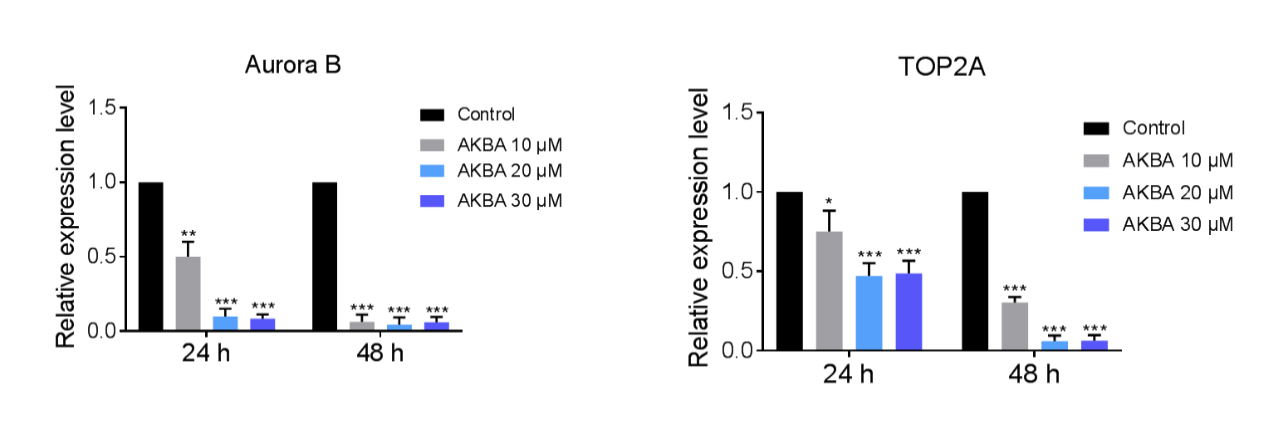
Figure S8.** **Quantification of the protein expression levels of Aurora B and TOP2A in U251 cells after the treatment of AKBA.** Data were presented as mean ± SD, * P < 0.05, ** *P* < 0.01, ****P* < 0.0001 vs. control group.

**
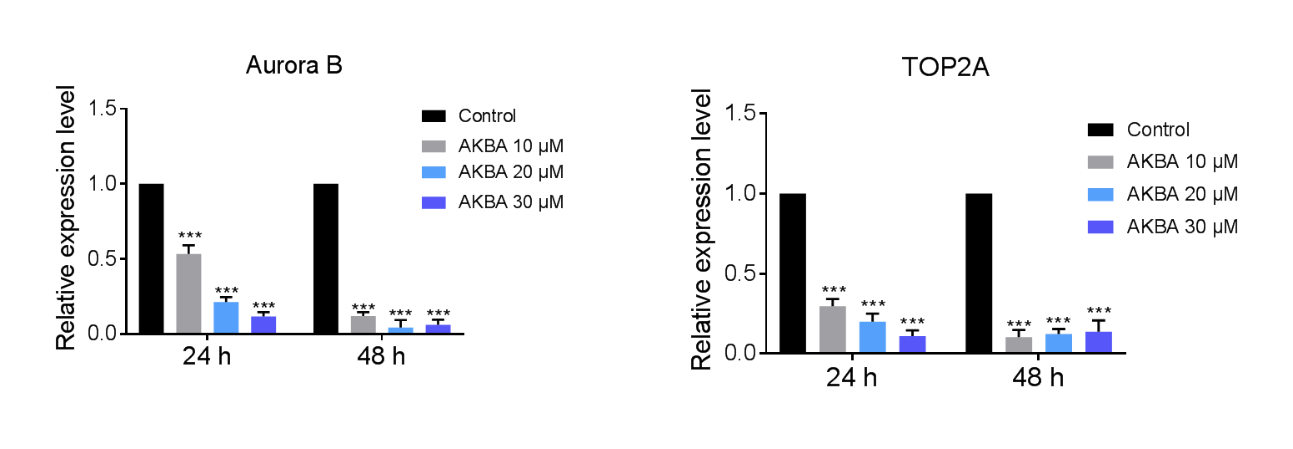
**

**Figure S9. Quantification of the protein expression levels of Aurora B and TOP2A in U87-MG cells after the treatment of AKBA.** The experiments were performed in triplicate, and the data were presented as mean ± SD, ****P* < 0.0001 vs. control group.
